# Facilitating improvements in young people’s social relationships to prevent or treat depression: there’s an app for that

**DOI:** 10.1038/s41398-021-01597-z

**Published:** 2021-09-09

**Authors:** Bridianne O’Dea

**Affiliations:** Black Dog Institute, Faculty of Medicine, University of New South Wales, Sydney, New South Wales 2031 Australia

**Keywords:** Scientific community, Human behaviour

**Dear Editor**,

I am writing in support of Filia and colleagues’ recent narrative review on empirically supported interventions that aim to prevent or treat depression in young people by targeting their relationships [1]. I agree with the central findings of this review, which assert that relationships present an effective therapeutic target for preventing and treating depression in youth. The review reported that young people preferred online approaches due to the accessibility and destigmatised nature of internet technology, particularly for minority groups and reluctant help-seekers. Notably, the authors exposed a disconnect between the therapy approaches young people considered important for relationships and those that have been evaluated. I am therefore writing to alert readers to a recent trial of a relationship-focussed mobile application (app) that aimed to overcome this disconnect, published only 19 days after the literature search was completed by Filia and colleagues [1]. While this recent trial would not have satisfied the full inclusion criteria of the narrative review, I believe the novelty of the intervention and the associated findings provide further support for Filia and colleagues’ conclusions.

In this recent trial, researchers and clinicians from the Black Dog Institute partnered with young people to develop a brief, self-directed, universal mobile app that aimed to improve mental health by targeting relationships [2]. Based on the principles of Cognitive Behavioural Therapy and Social Learning Theory, the WeClick app delivered a range of cognitive activities and behavioural strategies to reduce negative thinking about oneself and relationships [3]. The app also used social learning to demonstrate the positive outcomes of help-seeking for relationship problems [4, 5]. A two-arm randomised controlled trial among 193 young people (aged 12–16 years) demonstrated that the app was effective for improving wellbeing and help-seeking intentions for mental health, relative to the wait-list control [6]. No significant effects were found for depression; however, the study was not adequately powered to detect these. The trial confirmed the demand, interest and high degree of openness towards mobile apps for improving relationships: Over 1300 young people expressed interest in the app and over two-thirds completed all content. The rates of uptake, completion and retention were higher than many other self-directed online therapy programs for youth [7], where adherence remains a key challenge. This supports Filia and colleagues’ assertion that mobile devices may be superior to online interventions for sustaining engagement [8]. A high proportion of the sample also identified as LGBTQI+, supporting Filia and colleagues’ notion that digital approaches may have greater appeal to marginalised young people.

Further to the conclusions of Filia and colleagues’ review, we argue that mobile apps can provide immediate access, support and resources to young people when they are most in need. In this way, a brief, accessible, relationship-focussed intervention like WeClick may provide a highly acceptable and effective approach to improving the mental health of youth. We hope the field is encouraged by these positive findings and that we continue to embrace the potential of mobile apps for improving mental health outcomes in young people.

Yours respectfully,

Dr. Bridianne O’Dea, PhD
